# Axillary-iliac bypass to protect a transplanted kidney during abdominal aortic aneurysm repair: case report

**DOI:** 10.1590/1677-5449.200082

**Published:** 2021-05-31

**Authors:** Paula de Oliveira Trintinalha, Lucas Mansano Sarquis, Wilson Michaelis, Antonio Lacerda Santos, Rogerio Akira Yokoyama, Marcelo Tizzot Miguel, Adriana Pires Smaniotto, Mariana Santos de Oliveira

**Affiliations:** 1 Hospital do Trabalhador, Curitiba, PR, Brasil.; 2 Hospital Universitário Evangélico Mackenzie, Curitiba, PR, Brasil.

**Keywords:** abdominal aortic aneurysm, kidney transplantation, vascular surgical procedures

## Abstract

Abdominal aortic aneurysms (AAA) are the most common type, even when compared to those involving other segments of the aorta. The prevalence and natural history of arterial aneurysms in abdominal organ transplant recipients remain uncertain. We report a case of abdominal aortic aneurysm in a kidney transplant patient with contrast allergy. Conventional abdominal aortic aneurysm repair was performed, constructing a bi-iliac aortic bypass. A temporary bypass was constructed from the right axillary artery to the right common iliac artery to maintain the renal graft. The patient was transferred to the intensive care unit, where he remained hemodynamically stable, and he was discharged on the 2nd postoperative day. Conventional open surgery with temporary extra-anatomic bypass is an alternative option for treatment of AAA in patients with transplanted kidneys.

## INTRODUCTION

Abdominal aortic aneurysms (AAA) are the most frequent type, even when compared to those involving other segments of the aorta.^1^ Cases of AAA in kidney transplant patients are rare, but improved survival of transplant patients combined with the association of cardiovascular disease with kidney disease have led to increasing incidence of AAA in this group of patients.^2^^-^4

The prevalence and natural history of arterial aneurysms in recipients of abdominal organ transplants remain uncertain,^5^ but these patients have certain peculiarities that should be considered in the event that arterial aneurysm repair is needed: ongoing immunosuppression, the need to protect the graft, and the implications of aortic clamping.^4^ Techniques to minimize the effects of renal ischemia have been described, including extra-anatomic shunts, cooling of the graft, and extracorporeal circulation.^3^ The objective of this article is to describe management of a case of AAA in a kidney transplant patient. The Research Ethics Committee approved this study (decision number 4.192.559).

## CASE DESCRIPTION

**Figure 1:**
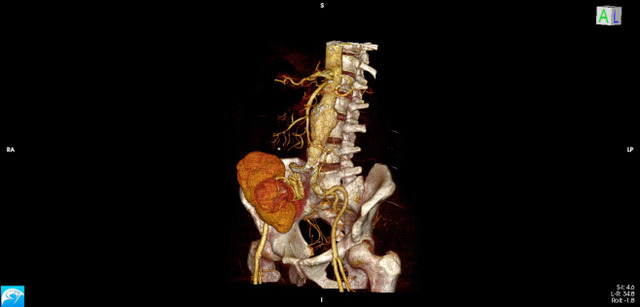
Angiotomography reconstruction showing the abdominal aortic aneurysm and the renal graft.

The patient was a 53-year-old male who had received a kidney transplant 12 years previously from a live donor. The underlying cause of his kidney disease was unknown and he was in follow-up at a Lymphedema and Angiodysplasia Clinic for an abdominal aortic aneurysm. He was asymptomatic. Physical examination found pulses present in all segments of the upper and lower limbs, a flaccid abdomen, and a pulsating mass and the renal graft in the right iliac fossa. He had a history of allergy to iodinated contrast. He was on mycophenolate sodium, tacrolimus, prednisone, losartan, and simvastatin. He is an ex-smoker. Abdominal computed tomography angiography was performed after antiallergic preparation, showing a fusiform aneurysm of the infrarenal segment of the aorta, with mural thrombi. The aneurysm began 13 mm from the emergence of the right renal artery, had a proximal neck measuring 24 x 24 mm, maximum diameters of 53 x 50 mm, and extended for 85 mm, up to the bifurcation of the aorta. The renal graft and its anastomosis to the right external iliac artery were identified (Figure 1).

**Figure 2:**
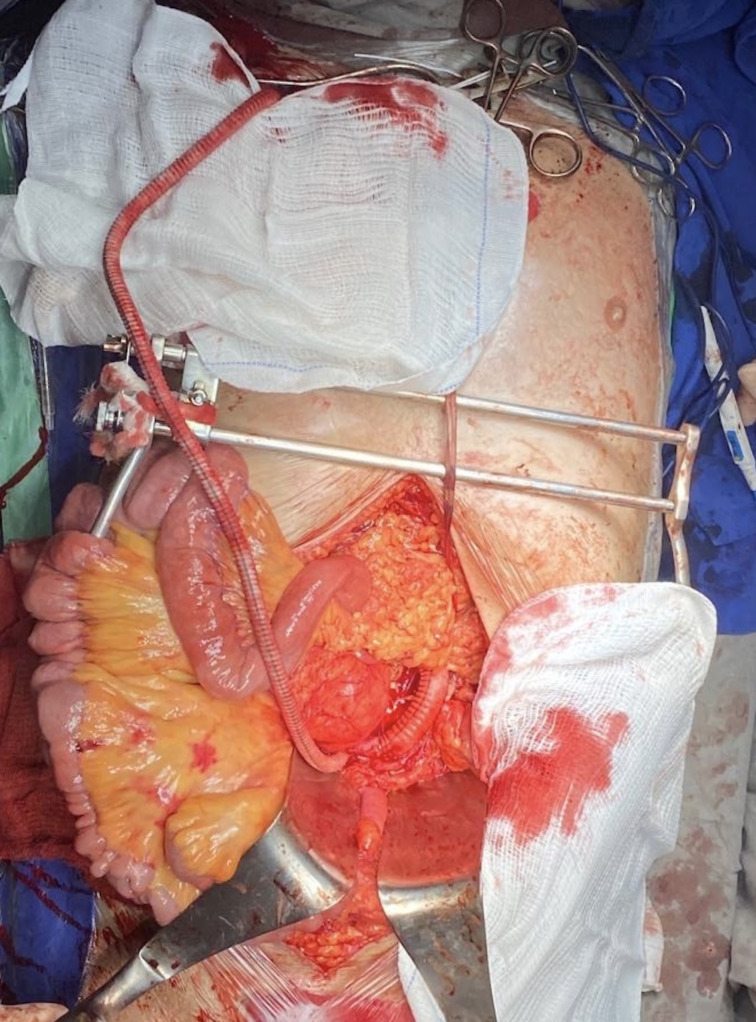
Right axillary to iliac bypass for vascularization of the renal graft. Bi-iliac aortic bypass after conventional treatment of abdominal aortic aneurysm.

The decision was taken to perform open surgical repair of the AAA. A temporary bypass was constructed from the right axillary artery to the right common iliac artery with a 7 x 60 mm straight Dacron graft to maintain perfusion of the renal graft while the aorta was clamped. Conventional repair of the abdominal aortic aneurysm was accomplished with a bi-iliac aortic bypass using a 14 x 7 mm bifurcated Dacron graft anastomosed distally to the common iliac arteries (Figure 2). The aorta was clamped infrarenally. The axillary-iliac graft was removed at the end of the procedure.

The patient was transferred to the intensive care unit, where he remained hemodynamically stable, and was discharged on the 2nd postoperative day. He had no motor dysfunction in any of his limbs. At 3 months’ follow-up the patient’s renal function is stable.

## DISCUSSION

Abdominal aortic aneurysms exhibit more aggressive behavior in transplant patients than in the general population, contradicting data that suggest immunosuppressant treatment reduces aneurysm growth.^3^ Immunomodulatory treatment after solid organ transplantation increases the risk of aneurysm rupture^4^ and expansion rates also increase after transplantation.^5^^,^^6^ As post-transplant survival continues to improve, the importance of diagnosis and adequate management of AAA increases.^5^

It is recommended that all AAA with diameters exceeding 5.0 cm should be repaired immediately and aneurysms with smaller diameters should be rigorously monitored (imaging every 6 months). Aortic aneurysms expand twice as fast in patients with abdominal organ or cardiac transplants than in patients who have not received transplants.^6^ In the present case, surgical treatment was indicated because of the diameter of the aneurysm and the increased risk of rupture due to the patient’s immunomodulatory treatment. Conventional treatment was chosen rather than an endovascular approach because of the patient’s history of allergy to iodinated contrast and the risk of nephropathy. One alternative to iodinated contrast would be to use carbon dioxide (CO_2_), but unfortunately this is not yet available on the Brazilian National Health Service (Sistema Único de Saúde) at our institution.

Surgical repair of aneurysms is complex in transplant patients because arterial support for the graft is generally supplied by the iliac artery, which is distal of the aneurysm.^3^ Renal grafts in this location can suffer ischemic damage during aortic surgery because the blood supply is suspended.^3^^,^^7^ Several techniques for maintaining renal perfusion have been described, including temporary extra-anatomic bypasses.^3^ In the present case, the strategy used to maintain renal perfusion was a bypass from the right axillary to the common iliac. We chose to make the distal anastomosis of this bypass to the iliac artery rather than the femoral artery to avoid an additional access via the groin, since the aneurysm was repaired using a bi-iliac aortic bypass.

There is no consensus on the ideal approach to protecting the renal graft during open AAA repair.^7^ Without the ischemia induced by clamping the aorta, endovascular repair would appear to be an alternative option. Short-term mortality after endovascular repair is clearly lower than after conventional surgery, although long-term reintervention rates are higher. Endovascular repair involves the risk of contrast nephropathy, but this risk is similar to in the general population.^3^

## CONCLUSIONS

Open conventional surgery with a temporary extra-anatomic bypass is one option for treatment of AAA in patients with kidney transplants.
