# Supplementary material for: Axillary-iliac bypass to protect a transplanted kidney during abdominal aortic aneurysm repair: case report
Source: J Vasc Bras. 2021 May 31;20:e20200082. doi: 10.1590/1677-5449.200082 (PMC8191185; doi:10.1590/1677-5449.200082)
Supplement: Translation

# Translation: Derivação axilo-ilíaco para proteção de rim transplantado durante a correção de aneurisma de aorta abdominal: relato de caso

**Authors:** Paula de Oliveira Trintinalha, Lucas Mansano Sarquis, Wilson Michaelis, Antonio Lacerda Santos, Rogerio Akira Yokoyama, Marcelo Tizzot Miguel, Adriana Pires Smaniotto, Mariana Santos de Oliveira

**Affiliations:** 1 Hospital do Trabalhador, Curitiba, PR, Brasil.; 2 Hospital Universitário Evangélico Mackenzie, Curitiba, PR, Brasil.

**Keywords:** aneurisma de aorta abdominal, transplante de rim, procedimentos cirúrgicos vasculares

## Abstract

Os aneurismas de aorta abdominal (AAA) são os mais frequentes, mesmo quando comparados a outros segmentos da aorta. A prevalência e a história natural de aneurismas arteriais em receptores de transplante de órgão abdominal permanecem incertas. Relatamos a abordagem de um caso de aneurisma de aorta abdominal em um paciente transplantado renal e com alergia ao contraste. Foi realizado o tratamento convencional do aneurisma de aorta abdominal com um *by-pass* aorto bi-ilíaco. Para manutenção do enxerto renal, foi confeccionado um *by-pass* temporário da artéria axilar direita até a artéria ilíaca comum direita. O paciente foi encaminhado para a unidade de terapia intensiva, onde permaneceu estável hemodinamicamente e recebeu alta no 2º pós-operatório. A cirurgia convencional aberta com derivação extra-anatômica temporária é uma alternativa para o tratamento do AAA em pacientes com transplante renal.

## INTRODUÇÃO

Os aneurismas de aorta abdominal (AAA) são os mais frequentes, mesmo quando comparados a outros segmentos da aorta^1^. A concomitância de AAA e transplantados renais é infrequente; entretanto, a melhora da sobrevida do paciente transplantado, aliada à correlação de doença cardiovascular com doença renal, mostra aumento da incidência de AAA nesse grupo de pacientes^2^^-^4.

A prevalência e a história natural de aneurismas arteriais em receptores de transplante de órgão abdominal permanecem incertas^5^, mas esses pacientes apresentam particularidades que devem ser consideradas para o eventual tratamento de aneurismas arteriais: vigência de imunossupressão, proteção do enxerto e clampeamento aórtico^4^. Técnicas para minimizar os efeitos da isquemia renal têm sido descritas, como derivações extra-anatômicas, resfriamento do enxerto ou circulação extracorpórea^3^. O objetivo deste estudo foi relatar a abordagem de um caso de AAA em um paciente transplantado renal. O protocolo foi aprovado pelo Comitê de Ética da nossa instituição (parecer número 4.192.559).

## DESCRIÇÃO DO CASO

Paciente do sexo masculino, 53 anos, transplantado renal há 12 anos, doador vivo, sem conhecimento da causa de base da doença renal, em seguimento no ambulatório de cirurgia vascular por aneurisma de aorta abdominal. Encontrava-se assintomático. Ao exame físico, pulsos presentes em todos os segmentos de membros inferiores e superiores, abdome flácido, com presença de massa pulsátil e enxerto renal em fossa ilíaca direita. Apresentou história de alergia ao contraste iodado. Em uso de micofenolato de sódio, tacrolimus, prednisona, losartana e sinvastatina. O paciente é ex-tabagista. A angiotomografia computadorizada de abdome, realizada após preparo antialérgico, visualizou aneurisma fusiforme do segmento infrarreal da aorta, com trombos murais, que se inicia 13 mm da emergência da artéria renal direita, com colo proximal de 24 x 24 mm, diâmetros máximos de 53 x 50 mm, e que se estende por 85 mm até a bifurcação da aorta. Foi identificada presença de enxerto renal com anastomose na artéria ilíaca externa direita (Figura 1).

**Figura 1:** Reconstrução angiotomográfica do aneurisma de aorta abdominal e do enxerto renal.

Optou-se por realização de tratamento do AAA através de cirurgia aberta. Com o objetivo de manter a perfusão do enxerto renal durante o clampeamento aórtico, foi confeccionado um *by-pass* temporário da artéria axilar direita até a artéria ilíaca comum direita (prótese de Dacron reta 7 x 60 mm). Realizado o tratamento convencional do aneurisma de aorta abdominal com um *by-pass* aorto bi-ilíaco (prótese de Dacron bifurcada 14 x 7 mm) com as anastomoses distais nas artérias ilíacas comuns (Figura 2). O clampeamento aórtico foi infrarrenal. Ao fim do procedimento, foi retirada a derivação axilo-ilíaco.

**Figura 2:** Derivação axilo ilíaco direito para vascularização do enxerto renal. Derivação aorto bi-ilíaca após tratamento convencional do aneurisma de aorta abdominal.

O paciente foi encaminhado para a unidade de terapia intensiva, onde permaneceu estável hemodinamicamente e recebeu alta no 2º pós-operatório. Não apresentou alteração motora de membros. Após 3 meses de seguimento, o paciente mantém função renal estável.

## DISCUSSÃO

O AAA nos pacientes transplantados apresenta comportamento mais agressivo do que na população geral, contradizendo alguns dados que sugerem que o tratamento imunossupressor reduz o crescimento do aneurisma^3^. A terapia imunomoduladora após transplante de órgãos sólidos aumenta o risco de ruptura do aneurisma^4^, além de apresentar maiores taxas de expansão após o transplante^5^^,^^6^. À medida que a sobrevida após o transplante continua a melhorar, o diagnóstico e o manejo adequado do AAA são cada vez mais importantes^5^.

Recomenda-se o reparo imediato de todos os AAA com diâmetro igual ou superior a 5,0 cm e vigilância rigorosa (imagens a cada 6 meses) para os aneurismas com menor diâmetro. Comparados aos pacientes sem transplante, os aneurismas da aorta em pacientes com transplantes de órgãos abdominais e cardíacos se expandem duas vezes mais rapidamente^6^. Neste caso, o tratamento cirúrgico foi indicado devido ao diâmetro do aneurisma, associado ao risco superior de rotura em virtude dos imunomoduladores utilizados pelo paciente. A opção pelo tratamento convencional em detrimento ao tratamento endovascular se deu pelo histórico de alergia ao contraste iodado, bem como pelo risco de nefropatia. A utilização de dióxido de carbono (CO2) é uma alternativa ao contraste iodado, mas, infelizmente, ainda não disponível em nosso serviço pelo Sistema Único de Saúde (SUS).

O reparo cirúrgico dos aneurismas em transplantados é complexo, pois o suporte arterial do enxerto geralmente deriva da artéria ilíaca, localizada distalmente ao aneurisma^3^. Essa localização do enxerto renal é passível de sofrer dano isquêmico durante a cirurgia aórtica, pois o suprimento sanguíneo para o enxerto renal é interrompido^3^^,^^7^. Várias técnicas para manter a perfusão renal foram relatadas, como as derivações extra-anatômicas temporárias^3^. No presente caso, a estratégia de manutenção da perfusão renal utilizada foi de uma derivação axilo-ilíaca comum direita. Optamos pela anastomose distal deste *by-pass* na artéria ilíaca e não na artéria femoral, para evitar um novo acesso a região inguinal, já que a correção do aneurisma foi realizada com uma ponte aorto bi-ilíaca comum.

Não há consenso sobre o manejo adequado para a proteção do enxerto renal durante o reparo aberto do AAA^7^. Sem isquemia induzida por clampeamento aórtico, a correção endovascular parece ser uma alternativa. A mortalidade a curto prazo do reparo endovascular é claramente inferior à cirurgia convencional, apesar de mostrar uma maior taxa de reintervenção à longo prazo. O reparo endovascular apresenta o risco de nefropatia por contraste, entretanto, o risco é semelhante ao da população em geral^3^.

## CONCLUSÃO

A cirurgia convencional aberta com derivação extra anatômica temporária é uma alternativa para o tratamento do AAA em pacientes com transplante renal.
